# Supplementary material for: Structural basis for inhibition of an archaeal CRISPR–Cas type I-D large subunit by an anti-CRISPR protein
Source: Nat Commun. 2020 Nov 25;11:5993. doi: 10.1038/s41467-020-19847-x (PMC7689449; doi:10.1038/s41467-020-19847-x)
Supplement: Supplementary file 1 — Supplementary Information [file 41467_2020_19847_MOESM1_ESM.pdf]

Structural basis for inhibition of an archaeal CRISPR-Cas type I-D large subunit by an anti-CRISPR protein

Manav et al.

Supplementary Information:

Supplementary Fig. 1-8

Supplementary Tables 1-2

Supplementary References

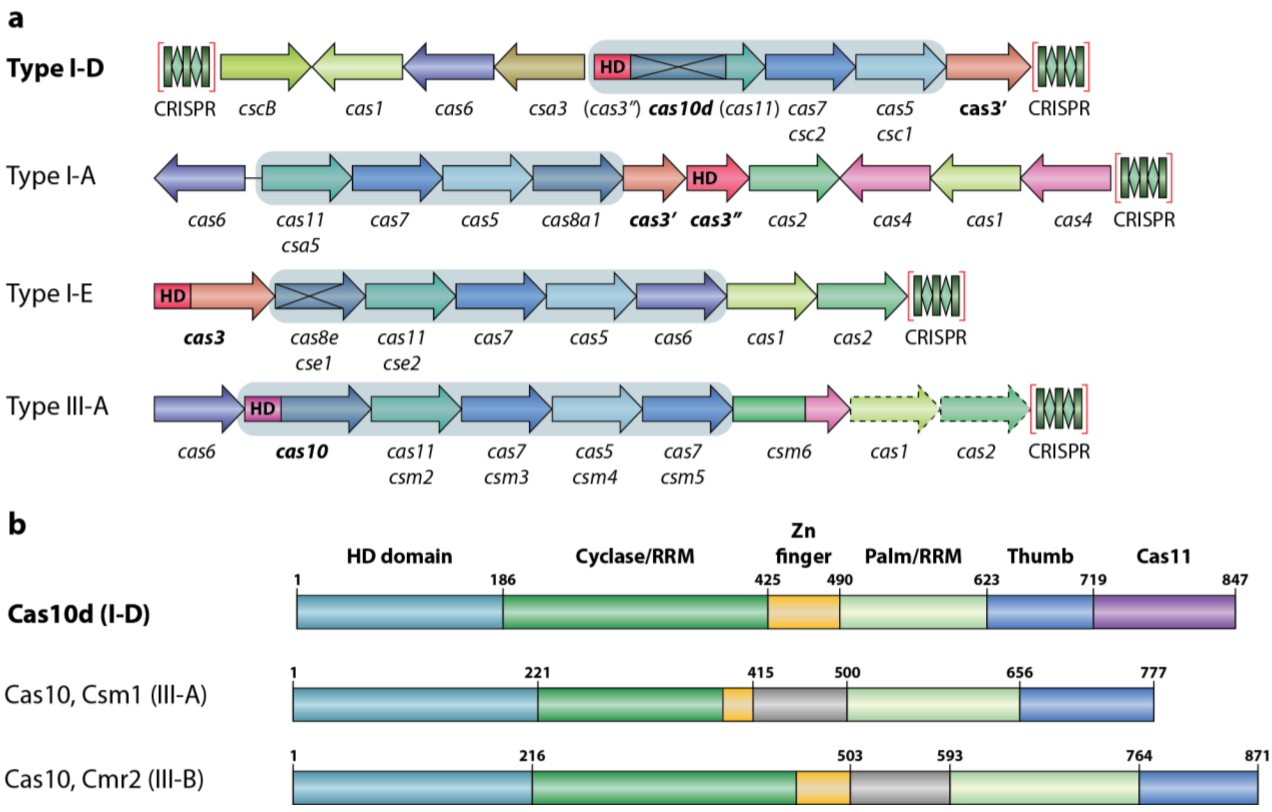

Supplementary Fig. 1. Organisation of relevant CRISPR-Cas loci and the domain structure of Cas10. a. Overview of the gene structure of the type I-D CRISPR-Cas locus found in *S. islandicus* (top, bold type) as compared to those of types I-A, I-E, and III-A based on the most recent classification of CRISPR-Cas systems with the effector complex members shown on a shaded background.[1] In type I-D, the large subunit, Cas10d, contains a type I HD domain similar to Cas3" (red), an inactivated polymerase domain, similar to type III Cas10 large subunits (dark blue, shown with a cross), and a C terminus with predicted homology to Cas11 (teal). In types I-A and I-E, the HD nuclease and SF2 helicase domains are encoded separately from the effector complex, either as *cas3* (type I-E) or as a split *cas3* gene structure (*cas3'* and *cas3"*, type I-A). In type III-A, the Cas10 (Csm1) large subunit forms part of the effector complex and includes a HD domain different from that in Cas3 (purple) followed by several polymerase domains. Note that Cas11 is encoded just downstream of Cas10. **b.** Overview of the domain structure of Cas10d (type I-D, bold type, this work) compared to Cas10 proteins from types III-A (Csm1) and III-B (Cmr2). Residue numbers at domain borders are shown, based on the structures of *T. onnurineus* Csm1 (PDB ID 4UW2) and *P. furiosus* Cmr2 (PDB ID 4W8Y).[2, 3]

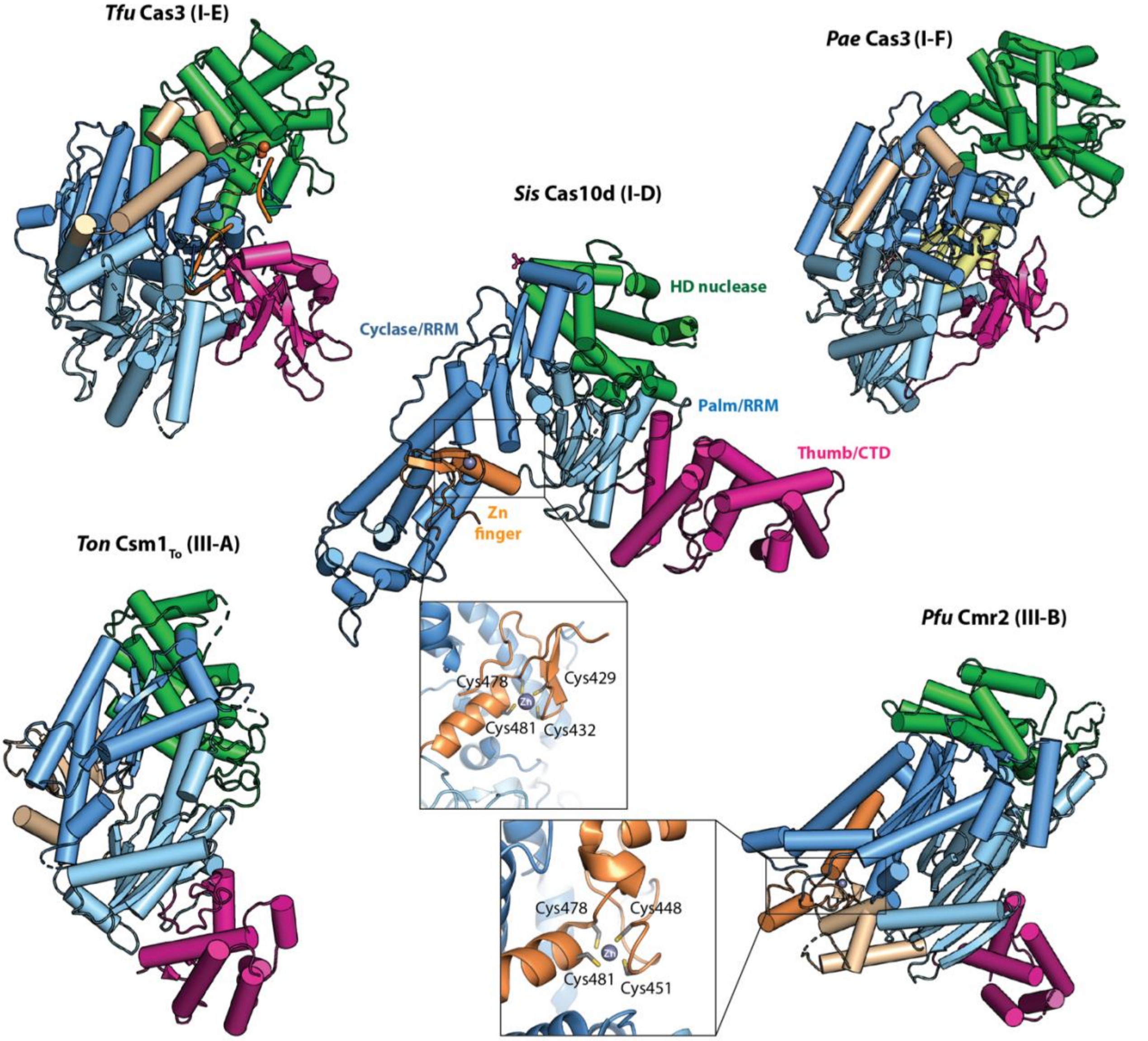

Supplementary Fig. 2. Comparison of known Cas3 and Cas10-like structures. Overview of the overall structure and domain organisation of *T. fusca* Cas3 (type I-E, PDB ID 4QQW, top left)[4], *P. aeruginosa* Cas3 (type I-F, PDB ID 5B7I, top right)[5], *S. islandicus* Cas10d (type I-D, this work, centre), *T. onnurineus* Cas10 (Csm1, type III-A, PDB ID 4UW2, bottom left)[3], and *P. furiosus* Cas10 (Csm2, type III-B, PDB ID 4W8Y).[2] Domain colours are: HD nuclease (green), cyclase/RRM (dark blue), zinc finger (orange), palm/RRM (light blue), and thumb/C-terminal domain (magenta). The two insets show the details of the Zn^{2+} binding sites found in Cas10d and Cmr2.

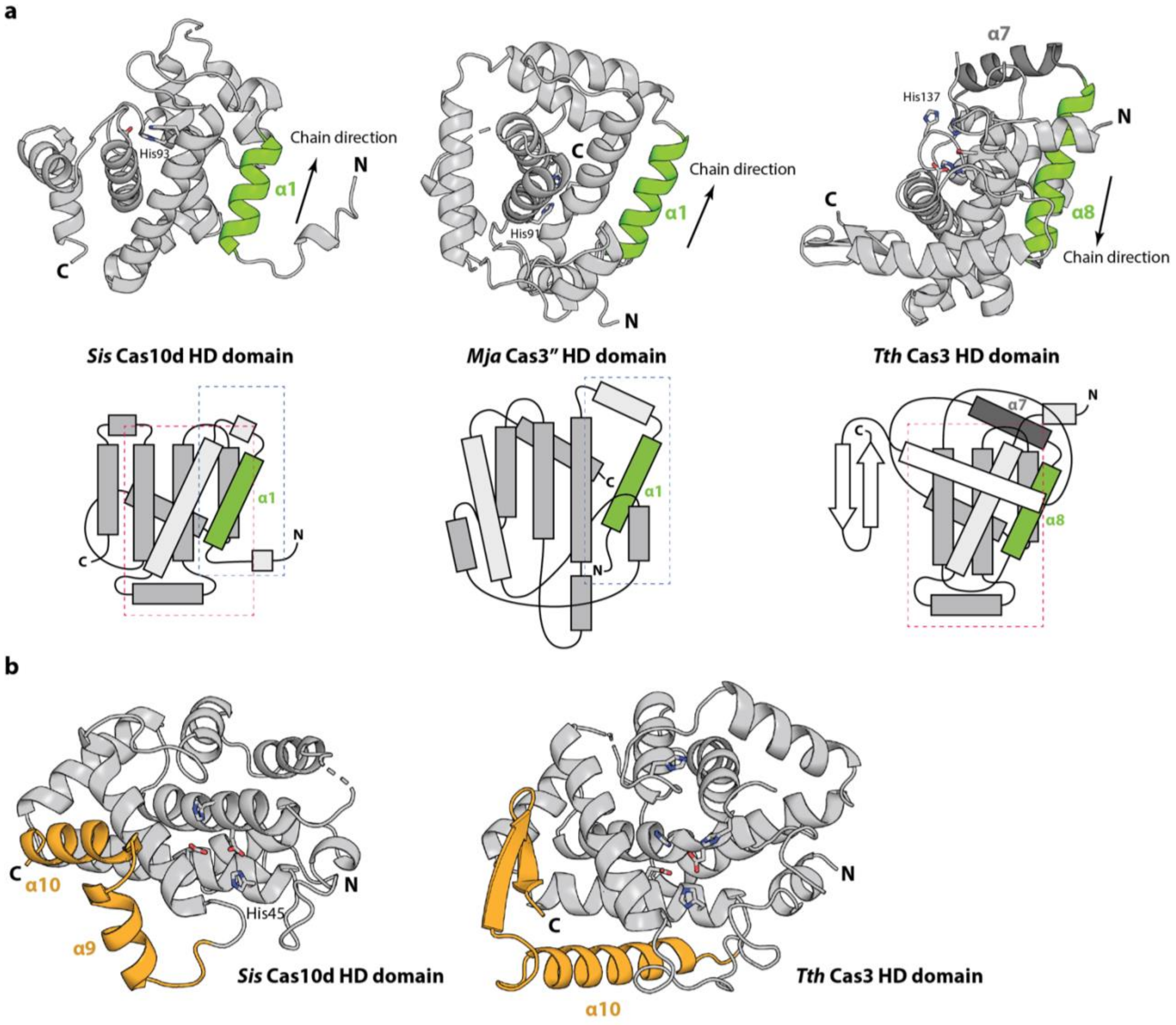

Supplementary Fig. 3. Details of the Cas10/Cas3 HD nuclease domains. a. Comparison of the location and orientation over the overlapping α1/α8 in the Cas3 and Cas3" HD domains shown as cartoons (top) and topology diagrams (bottom). Left, structure of the *S. islandicus* Cas10d HD domain (this work) with the N-terminal helix shown in green and chain direction indicated. The location of the active site and His93 is shown for comparison; Middle, structure of the *M. jannaschii* Cas3" HD domain (PDB ID 3S4L) showing the identical arrangement of the N-terminal helix, but deviations in the core domain, including location of the active site[6]; Right, structure of the *T. thermophilus* Cas3 HD domain (PDB ID 3SK9) with the structurally equivalent helix α8 shown in green.[7] Note the difference in chain direction for α8 but overall similar core fold to Cas10d, including active site location. Regions bridging to and from helix α8 from the core domain fold, including helix α7, are shown in darker grey. In the topology diagrams, the N-terminal structural elements that are shared

between the *S. islandicus* (left) and *M. jannaschii* (middle) HD domains are highlighted with a blue, dashed box while the core fold shared between the *S. islandicus* (left) and *T. thermophilus* (right) HD domains is highlighted with a red box. **b.** Comparison of the C-terminal elements of the HD domains found in *S. islandicus* Cas10d (this work, left) and the *T. thermophilus* Cas3 HD domain (PDB ID 3SK9, right).[7] Helices α9-α10 of Cas10d (shown in orange) spatially occupy the same region as helix α10 and a short β-hairpin in Cas3.

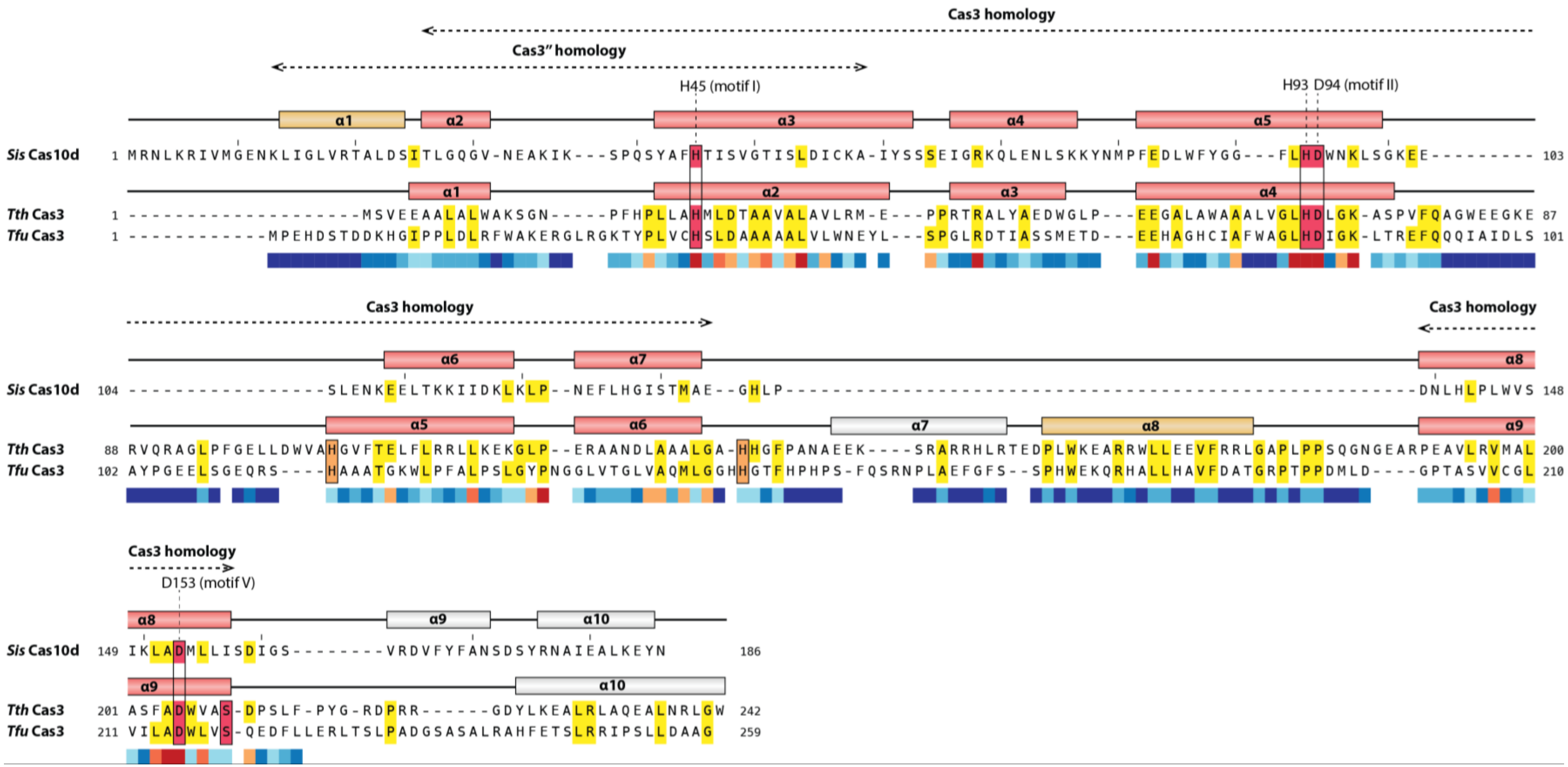

Supplementary Fig. 4. Sequence alignment of Cas10d and Cas3 HD nuclease domains. Alignment of the sequences of the HD domains of *S. islandicus* (*Sis*) Cas10d (this work, residues 1-186), *T. thermophilus* (*Tth*) Cas3 (residues 1-242, based on PDB ID 3SK9)[7], and *T. fusca* (*Tfu*) Cas3 (residues 1-259, based on PDB ID 4QQW).[4] Conserved residues are shown on yellow background with overall conservation indicated by the coloured bar from not conserved (dark blue) to fully conserved (red). Secondary structure elements observed in Cas10d and *Tth* Cas3 are shown above their respective sequences, where red helices indicate the shared helical core, yellow the swapped, conserved helix (see text), and grey non-conserved elements. Regions of structural homology to Cas3" (based on the structure of the *M. jannaschii* Cas3 nuclease MJ0384, PDB ID 3S4L)[6] and Cas3 (based on *Tth* Cas3) are shown with dashed arrows.

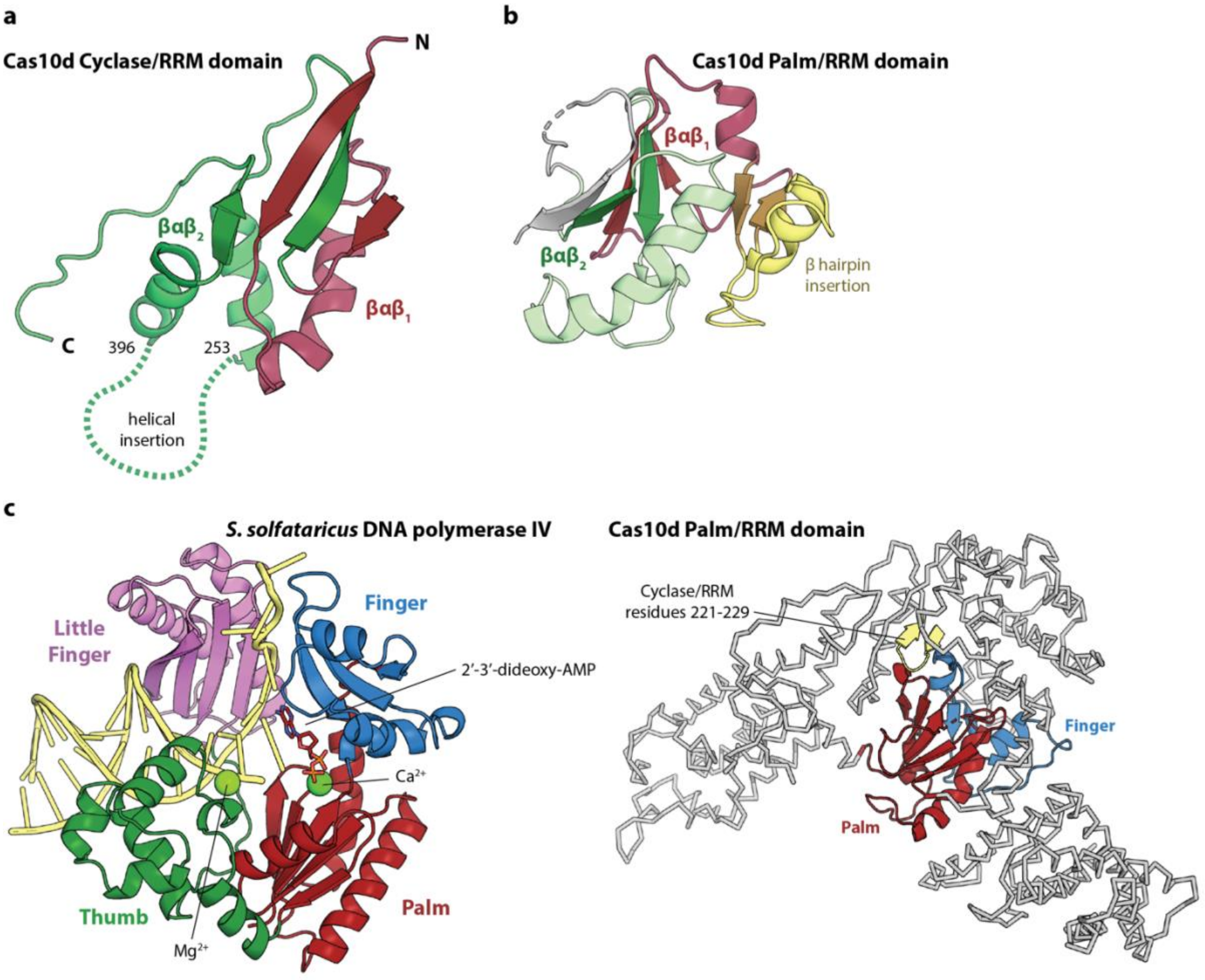

Supplementary Fig. 5. Details of the Cas10d RRM domains. a. Details of the Cas10d Cyclase/RRM (RRM1) domain. The two interleaved βαβ units of the RRM domain are shown in red and green colours, respectively. The second unit has a large helical domain of almost 150 residues inserted into the RRM fold between the two strands. **b.** Details of the Palm/RRM (RRM2) domain with colours as in A. The first βαβ unit (green) has a unique β hairpin insertion (yellow). **c.** Comparison of the Palm domain as found in *S. solfataricus* DNA polymerase IV (Dpo4, PDB ID 1JX4, left) with the Palm/RRM domain in Cas10d (right). The Palm domain is in red and shown in similar orientations in the two figures. For DNA polymerase IV, the locations of divalent metal ions (Ca^{2+} and Mg^{2+}) as well as incoming nucleotide (2'-3'-dideoxy ADP) are indicated and DNA is shown as a yellow ribbon. The finger domain (β hairpin insertion) located after the first sheet of the Palm domain is shown in blue and is found in two different orientations. Cas10d lacks the residues required for coordination of a metal ion in the active site, and another hairpin loop (residues 221-229, yellow) in the Cyclase/RRM domain blocks the site where a nucleotide would bind.

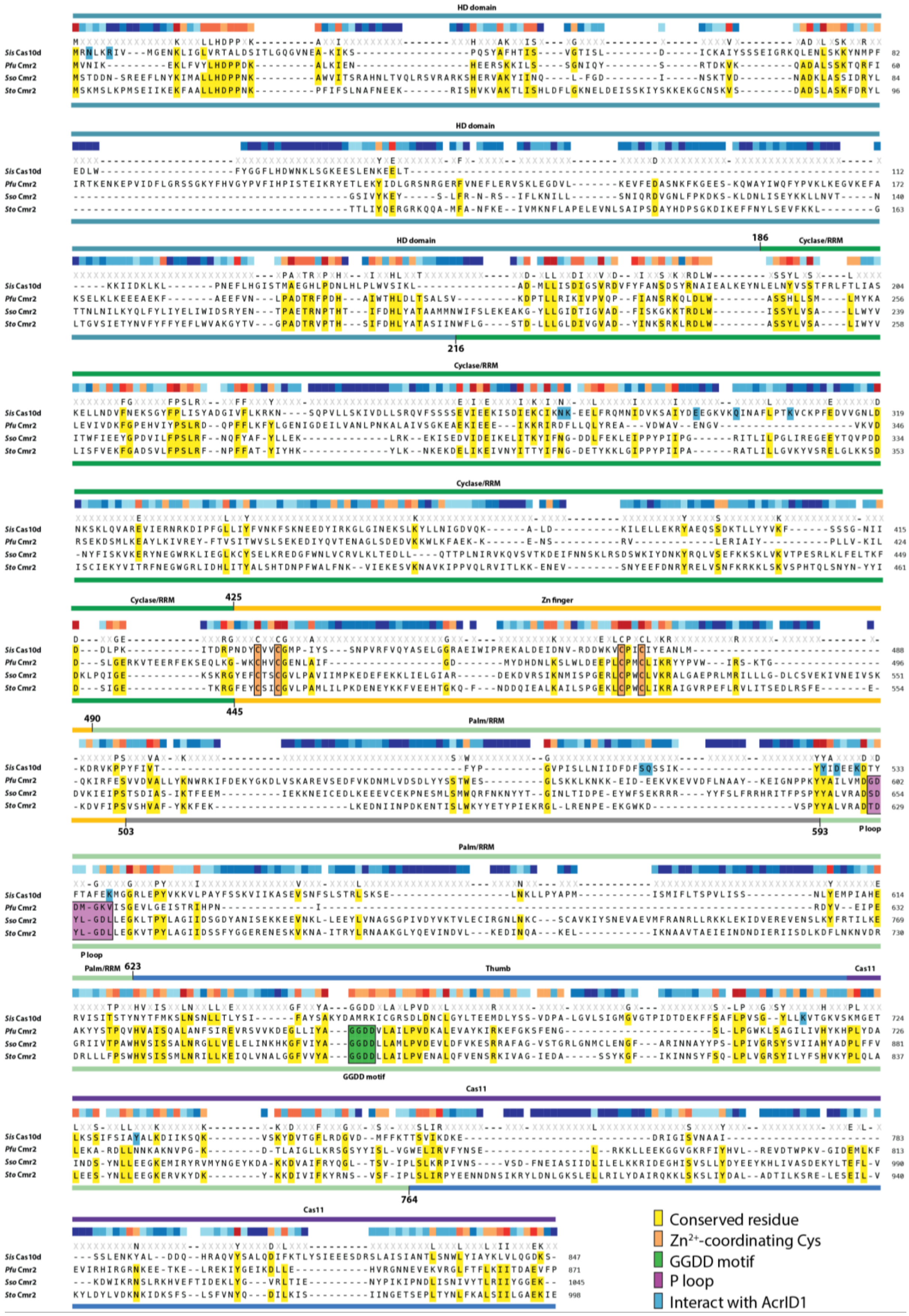

Supplementary Fig. 6. Alignment of Cas10d to members of the type III Cmr2 family.

Sequence alignment of *S. islandicus* Cas10d (*Sis*, this work) to the sequences of Cmr2 proteins from *P. furiosus* (*Pfu*, UniProt Q8U1S6), *S. solfataricus* (*Sso*, Q97WX0), and *S. tokodaii* (*Sto*, Q96Z52). Conserved residues are shown on yellow background with overall conservation indicated by the coloured bar from not conserved (dark blue) to fully conserved (red). Domains in Cas10d are indicated with coloured and labelled bars above the sequences, while those in Cmr2 (based on the structure of *Pfu* Cmr2) are shown with bars below the sequences.[8] The four cysteines involved in Zn^{2+} binding in the zinc finger motif are shown on an orange background, and the P loop (consensus GXXXXGK[S/T]) and GGDD motifs are shown on purple and green backgrounds, respectively. Residues that interact with AcrID1 are shown on a light blue background.

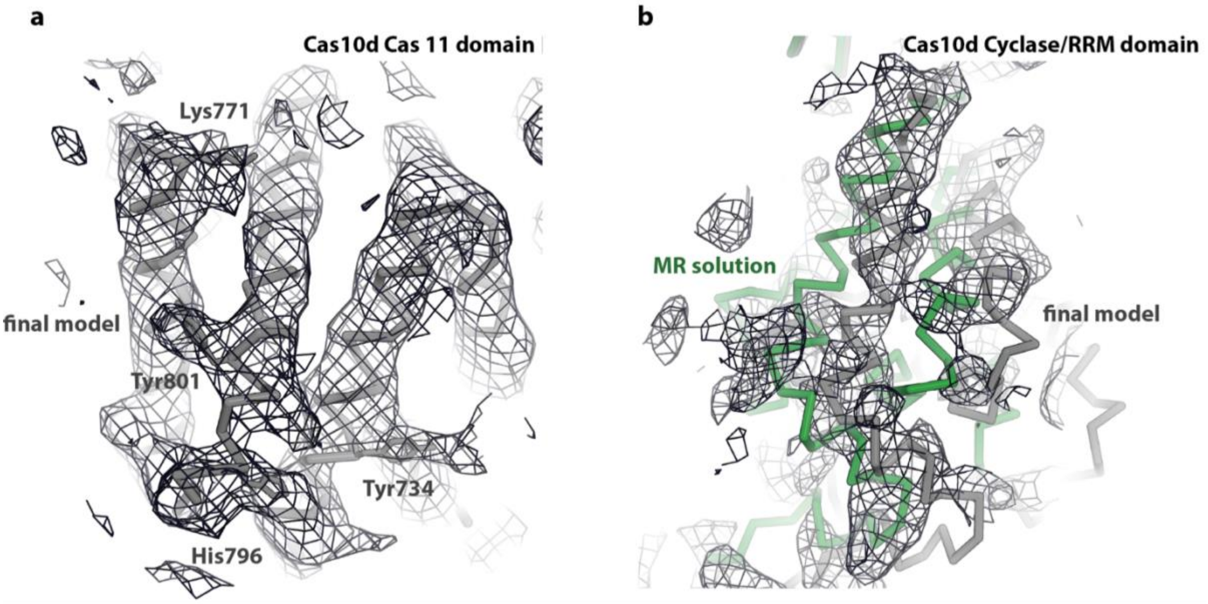

Supplementary Fig. 7. Electron density for isolated Cas10d at 4 Å resolution. a. Initial $2mF_o$-DF_c map from molecular replacement (Phenix.phaser) for a rigid part of the structure (Cas11-like domain, residues 719-847), contoured at 0.8 σ with the final, refined model and several large side chains in sticks. **b.** Initial $2mF_o$-DF_c map from molecular replacement (MR) for the flexible RRM/Cyclase domain (residues 490-623), contoured at 0.8σ. The green ribbon shows the (incorrect) initial MR model, while the final model is in grey.

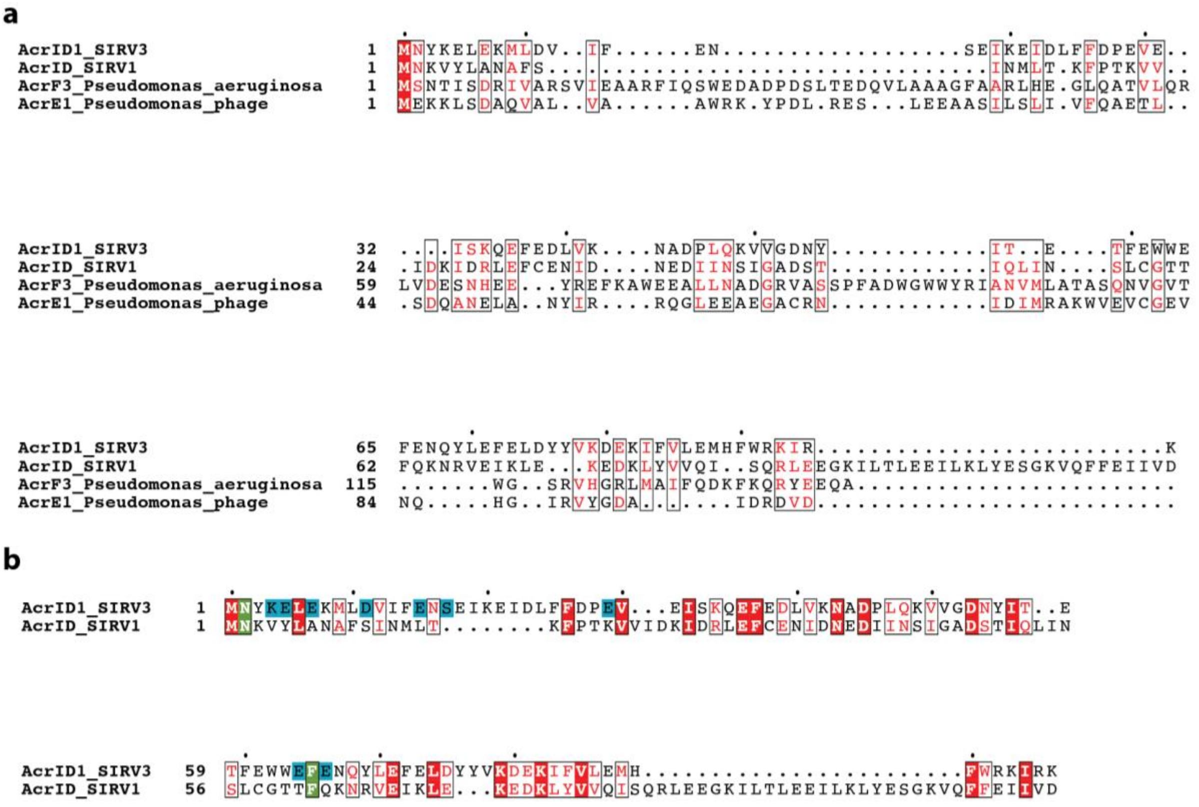

Supplementary Fig. 8. Alignment of anti-CRISPR protein sequences. a. Alignment anti-CRISPR (Acr) proteins targeting type I-D (AcrID1_SIRV3 and AcrID_SIRV1) and I-F CRISPR-Cas systems (*P. aeruginosa* AcrF3 and *Pseudomonas* phage AcrE1) showing their divergence. Conserved residues are shown in red. **b.** Alignment of the closely related type I-D targeting Acrs from *S. islandicus* phages SIRV1 and SIRV3. Conserved residues are shown on red background while those interacting with Cas10d are on blue background. Conserved interactions are on green background (only Asn2 and Phe65).

Supplementary Table 1. Crystallographic data statistics.

	Cas10d-AcrID1 (SeMet)	Cas10d (native, isolated)
**PDB ID**	**6THH**	**6YES**
Data Collection		
Wavelength (Å)	0.9793	0.9763
Resolution range	62.1 - 3.48 (3.60 - 3.48)*	91.3 - 4.00 (4.14 - 4.00)*
Space group	$P4_32_12$	$P4_32_12$
Unit cell dimensions		
a, b, c, (Å)	157.99, 157.99, 130.24	141.43, 141.43, 223.87
α, β, γ (°)	90, 90, 90	90, 90, 90
Total number of reflections	292,747 (29,185)	186,742 (19,221)
Unique reflections	21,713 (2139)	23,124 (1923)
Multiplicity	13.5 (13.6)	9.5 (10.0)
Completeness (%)	99.8 (99.7)	97.4 (85.5)
R_{merge} (%)	6.4 (68.5)	24.2 (335.2)
I/σ_I	11.9 (1.2)	5.6 (0.32)
$CC_{1/2}$ (%)	99.6 (55)	99.8 (16)
Refinement		
Average B-factor (Å^2)	137.1	228.3
No. of reflections	21,696 (2,134)	19,343 (1648)
No. of reflections (free)	1,868 (184)	929 (94)
R-work (%)	23.7 (35.6)	34.3 (47.4)
R-free (%)	26.6 (38.0)	37.0 (43.9)
No. of atoms		
protein	8,238	12,534
ligand	6	2
RMSD bonds (Å)	0.002	0.012
RMSD angles (°)	0.52	0.92
Ramachandran statistics (%)		
favoured	92.5	89.44
allowed	7.0	10.37
outliers	0.5	0.19
Phasing Statistics		
anomalous signal	4.2 (0.65) †	-
number of Se sites (theoretical)	21 (21)	-
figure of merit	0.32 / 0.52	-

* Numbers in parentheses indicate outermost resolution shell

† SIGANO, mean anomalous difference in units of its estimated standard deviation ($|F^+ - F^+|/\sigma_{|\Delta F|}$).

Supplementary Table 2. Nucleic acid oligos used in this study.

**Oligo**	**Sequence (5′-3′)**	**Description**
dsNTS target strand	TGTAAATCTATATCTAAAGCTTGTAAAACTATTTGAATATCTATAGATTGTTCTTTTCTCCTACTTTCCAAATTTCCACAGTCTGCATAATTTGTAGTTTTGTTAATTTTTTCCT	
dsNTS non-target strand	AGGAAAAAATTAACAAAACTACAAATTATGCAGACTGTGGAAATTTGGAAAGTAGGAGAAAAGAACAATCTATAGATATTCAAATAGTTTTACAAGCTTTAGATATAGATTTACA	The 5’ end of this strand was radio-labeled.

Supplementary References

1. Makarova KS, *et al.* Evolutionary classification of CRISPR-Cas systems: a burst of class 2 and derived variants. *Nat Rev Microbiol* **18**, 67-83 (2020).

2. Benda C, *et al.* Structural model of a CRISPR RNA-silencing complex reveals the RNA-target cleavage activity in Cmr4. *Mol Cell* **56**, 43-54 (2014).

3. Jung TY, An Y, Park KH, Lee MH, Oh BH, Woo E. Crystal structure of the Csm1 subunit of the Csm complex and its single-stranded DNA-specific nuclease activity. *Structure* **23**, 782-790 (2015).

4. Huo Y, *et al.* Structures of CRISPR Cas3 offer mechanistic insights into Cascade-activated DNA unwinding and degradation. *Nat Struct Mol Biol* **21**, 771-777 (2014).

5. Wang X, *et al.* Structural basis of Cas3 inhibition by the bacteriophage protein AcrF3. *Nat Struct Mol Biol* **23**, 868-870 (2016).

6. Beloglazova N, Petit P, Flick R, Brown G, Savchenko A, Yakunin AF. Structure and activity of the Cas3 HD nuclease MJ0384, an effector enzyme of the CRISPR interference. *EMBO J* **30**, 4616-4627 (2011).

7. Mulepati S, Bailey S. Structural and biochemical analysis of nuclease domain of clustered regularly interspaced short palindromic repeat (CRISPR)-associated protein 3 (Cas3). *J Biol Chem* **286**, 31896-31903 (2011).

8. Cocozaki AI, *et al.* Structure of the Cmr2 subunit of the CRISPR-Cas RNA silencing complex. *Structure* **20**, 545-553 (2012).
